# On the Potential of Time Delay Neural Networks to Detect Indirect Coupling between Time Series

**DOI:** 10.3390/e22050584

**Published:** 2020-05-21

**Authors:** Riccardo Rossi, Andrea Murari, Pasquale Gaudio

**Affiliations:** 1 Department of Industrial Engineering, University of Rome “Tor Vergata”, via del Politecnico 1, 00100 Roma, Italy; gaudio@ing.uniroma2.it; 2 Consorzio RFX (CNR, ENEA, INFN, Universita di Padova, Acciaierie Venete SpA), Corso Stati Uniti 4, 35127 Padova, Italy; andrea.murari@euro-fusion.org

**Keywords:** time series, indirect coupling, time delay neural networks, Lorenz system

## Abstract

Determining the coupling between systems remains a topic of active research in the field of complex science. Identifying the proper causal influences in time series can already be very challenging in the trivariate case, particularly when the interactions are non-linear. In this paper, the coupling between three Lorenz systems is investigated with the help of specifically designed artificial neural networks, called time delay neural networks (TDNNs). TDNNs can learn from their previous inputs and are therefore well suited to extract the causal relationship between time series. The performances of the TDNNs tested have always been very positive, showing an excellent capability to identify the correct causal relationships in absence of significant noise. The first tests on the time localization of the mutual influences and the effects of Gaussian noise have also provided very encouraging results. Even if further assessments are necessary, the networks of the proposed architecture have the potential to be a good complement to the other techniques available in the market for the investigation of mutual influences between time series.

## 1. Introduction to Indirect Coupling between Time Series

The task of detecting a mutual influence between time series remains a serious challenge in the analysis of complex systems [1]. Traditional correlation analysis has proven to be completely inadequate. Therefore, in the last few decades, various techniques have been devised to obtain more reliable results. Among the most successful are Granger causality [2], transfer entropy [3], recurrence analysis [4], and cross mapping [5]. Even if these methodologies have provided very interesting results, they all have their limitations. They can have some difficulties already in bivariate analysis, but their main deficiencies become evident when indirect coupling is to be extracted from the time series. The interactions between three different systems, particularly in the non-linear and chaotic regimes, often present a very significant challenge to each of these methods. Indeed, typically, they tend to work much better for a subset of problems [6]. By contrast, many efforts have recently been devoted to the investigation of neural networks of complex topologies [7]. It is therefore natural to ask the question whether this technology can also help in this field of causality detection for the time series and provide a good alternative to already established solutions.

Unfortunately, the assumption behind the architecture and training of traditional feed-forward neural networks is that all inputs (and outputs) are independent of each other. Of course, in many applications, this is a quite significant and unrealistic limitation. When causality relations have to be detected, the time evolution of systems becomes an essential aspect of the analysis. In this perspective, time delay neural networks (TDNNs) constitute a natural extension of traditional feed-forward neural networks because they are designed to learn from the past. In other words, TDNNs have a “memory” about what has been previously calculated. Moreover, the topology of TDNNs is also the most suited to our approach to the investigation of causal relationships between time series, as will be discussed in detail in the next section.

It is worth mentioning that the concept of causality, adopted in this work, is the one proposed by Wiener and based on predictability [8]. A time series is considered to have a causal influence on another, called target, if it contains information, which helps to predict the evolution of the target. Therefore, all the cases of influence and couplings considered in the present paper are to be interpreted in this sense of increased predictability.

**Figure 1:**
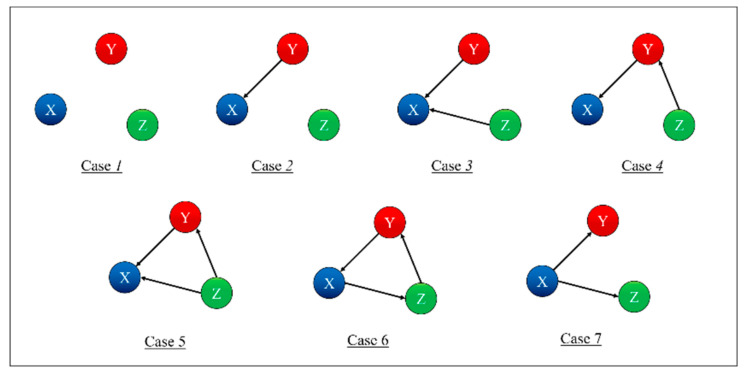
Simple networks showing the seven trivariate cases of mutual influence between three systems.

To test and prove the potential of TDNNs, they have been applied to the detection of indirect coupling. A systematic analysis of all possible cases involving three systems has been performed; the seven possible interrelations between three systems are represented as simple networks in Figure 1 [9]. The paper reports in detail the results obtained applying TDNNs to these cases and is structured as reported in the following. The family of recurrent networks implemented is overviewed in Section 2, together with the mathematical structure of the non-linear systems investigated, three coupled Lorenz systems. The main results of the numerical tests performed are the subject of Section 3. Section 4 analyses the issue of non-stationarity and time resolution. The first investigation of the effects of the noise is reported in Section 5. The summary and future developments are given in Section 6.

## 2. Time Delay Neural Networks and Coupled Lorenz Systems

**Figure 2:**
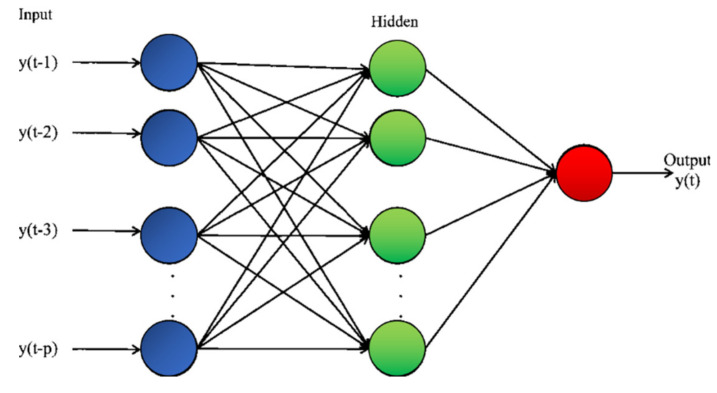
Topology of a time delay neural network of order *p*.

As mentioned, the future evolution of many systems can depend not only on their present state but also on their past. Moreover, even the behavior of systems, which do not strictly present memory effects, can be more easily learned by considering their evolution in time. Time delay neural networks present the simplest architecture to take the past of a time series into consideration when trying to forecast its future [10]. This type of network receives not only a time slice of data but a sequence of subsequent time points as inputs, as shown in Figure 2. Basically, the input is a window of length *p* into the past, which in our application is used to predict the following item in the input time series. Mathematically, this type of network realizes a non-linear autoregressive model of order *p*. Given their simple architecture, the basic techniques used to train traditional feed-forward networks can be easily transferred to the TDNN.

**Figure 3:**
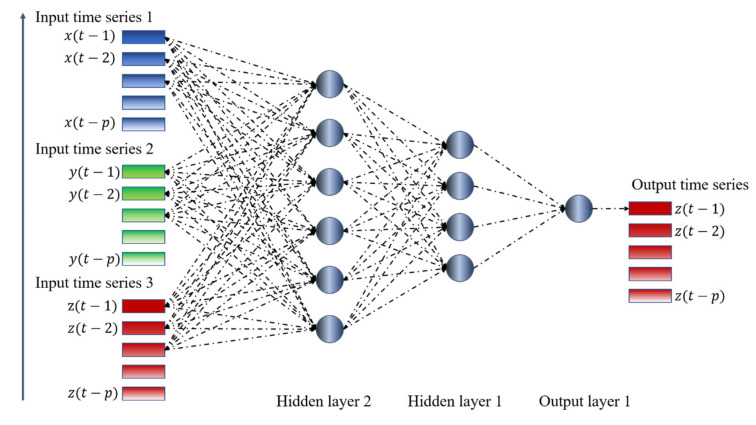
Architecture of the time delay neural networks used to investigate the indirect coupling between three systems.

By contrast, such a simple solution is not always adequate to learn the temporal structure of the data when the information required is about the coupling between systems. A slightly modified version of TDNNs, shown in Figure 3, is therefore the architecture adopted to perform the investigations reported in the rest of the paper. The TDNNs of this topology have been implemented with the MATLAB toolbox: The training technique is backpropagation implemented with the Levenberg–Marquardt algorithm. For the numerical cases described in the following, about 5000 epochs have proved to be normally sufficient (in any case, convergence has also been achieved for less than the maximum limit set to 10,000 epochs).

The topology reported in Figure 3 indeed allows investigating all the possible couplings of the systems, i.e., all the combinations shown in Figure 1. The proposed approach consists of training 12 different TDNNs to predict all the three-time series (of systems X, Y, and Z) for each of the seven cases shown in Figure 1. For each case and each time series to be predicted (X or Y or Z), four networks are deployed, with a different combination of the time series as input; one network is trained with all the three inputs, and the other three with one input removed each. For each case, the results are therefore 12 new time series, each one a prediction of the future evolution of one of the three systems, based on different combinations of inputs. The detection of the mutual influences is then based on the residuals, which are calculated first for the case, in which all the variables are used as inputs. Then, the residuals are also computed for the cases when one of the inputs is excluded from the input set. The smallest variance of the residuals, the one obtained considering all the inputs, is then compared with the ones of the other cases. If the variance of a certain output, obtained after removing an input, is statistically higher than the one calculated when all the inputs are included, then the removed quantity is considered to have a causal influence on that specific output.

To assess whether two variances of the residuals are statistically different, recourse has been made to the F-test [11]. The null hypothesis for this test is that of equality of variances. The null hypothesis of equal variances can be rejected at the desired statistical significance level. More importantly for our application, the test can provide the *p*-values to determine how unlikely the variance ratio is in case the null hypothesis is true. The *p*-values are the indicators used to assess the mutual influence between the various time series (see next section). They are converted into the probability of the null hypothesis of equal variance being true; if this probability is too low, the variables involved are considered causally related.

Following the treatment reported in [9], to test the potential and limitations of TDNNs, the mutual interactions of three coupled Lorenz systems X, Y, and Z in the chaotic domain have been investigated. Mathematically, the systems and their couplings are represented as follows:

System X:

(1) $$\frac{dx_{1}}{dt}=\sigma \left(x_{2}-x_{1}\right)$$

(2) $$\frac{dx_{2}}{dt}=rx_{1}-x_{2}-x_{1}x_{3}+\mu_{21}y_{2}^{2}+\mu_{31}z_{3}^{2}$$

(3) $$\frac{dx_{3}}{dt}=x_{1}x_{2}-bx_{3}$$

System Y:

(4) $$\frac{dy_{1}}{dt}=\sigma \left(y_{2}-y_{1}\right)$$

(5) $$\frac{dy_{2}}{dt}=ry_{1}-y_{2}-y_{1}y_{3}+\mu_{12}x_{2}^{2}+\mu_{32}z_{3}^{2}$$

(6) $$\frac{dy_{3}}{dt}=y_{1}y_{2}-by_{3}$$

System Z:

(7) $$\frac{dz_{1}}{dt}=\sigma \left(z_{2}-z_{1}\right)$$

(8) $$\frac{dz_{2}}{dt}=rz_{1}-z_{2}-z_{1}z_{3}+\mu_{13}x_{2}^{2}+\mu_{23}y_{2}^{2}$$

(9) $$\frac{dz_{3}}{dt}=z_{1}z_{2}-bz_{3}$$

The choice of the parameters is σ=10, r=28 e b=8/3. The coupling between the systems is modelled by the $\mu_{ij}$ coefficient, which is varied to simulate all the six situations described in the previous section. When $\mu_{ij}=0$ there is no mutual influence between the systems; a value different from zero indicates that the system *i* exerts an influence on system *j*.

## 3. Results of Coupling Detection

This section reports the results obtained for each case of coupling shown in Figure 1. In detail, the topology of the TDNNs deployed consists of two hidden layers, the first with six neurons and the second with four neurons, and one output layer of three neurons, equal to the number of time series considered. The TDNNs are trained to predict the following time point of all three-time series. The order of each input series is two, in the sense that only the two previous time points have been used. The number of points analyzed is 5000, a reasonable amount of data for the investigation of time series generated by non-linear systems. The inputs have then been divided in the training, validation, and test sets with a proportion of 70%/15%/15%.

*Case 1: Independent Systems*

(10) $$\mu_{21}=\mu_{31}=\mu_{12}=\mu_{32}=\mu_{13}=\mu_{23}=0$$

**Figure 4:**
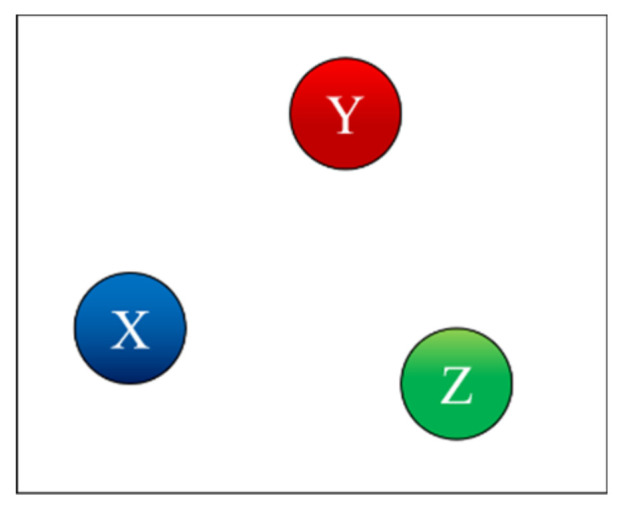
Coupling case 1.

Case 1 is the independent case, as shown in Figure 4. Table 1 indicates correctly that X depends only on X, Y depends only on Y, and Z depends only on Z. To interpret the values in the table, one should remember that for each row, i.e., for each predicted time series, the columns report the effect of removing the corresponding input to the TDNNs. Therefore, each entry of Table 1 shows the probability, calculated with the F-test, that the residual variances, when certain inputs of the TDNNs are suppressed, are not statistically different from the variances obtained by the networks using all inputs. Consequently, high values of the probabilities in the table mean that the probability of the null hypothesis (equal variance) being correct is also high. Of course, if, when removing an input, the TDNNs manage to reproduce a certain output series without a significant degradation in the variance or the residuals, then that specific input cannot have an appreciable causal influence on that specific output. Therefore, the high value of the entries in Table 1 and the following indicate that the corresponding systems do not present a significant causal relationship.

*Case 2: X is Influenced by Y*

(11) $$\mu_{21}=0.1;\;\mu_{31}=\mu_{12}=\mu_{32}=\mu_{13}=\mu_{23}=0$$

**Figure 5:**
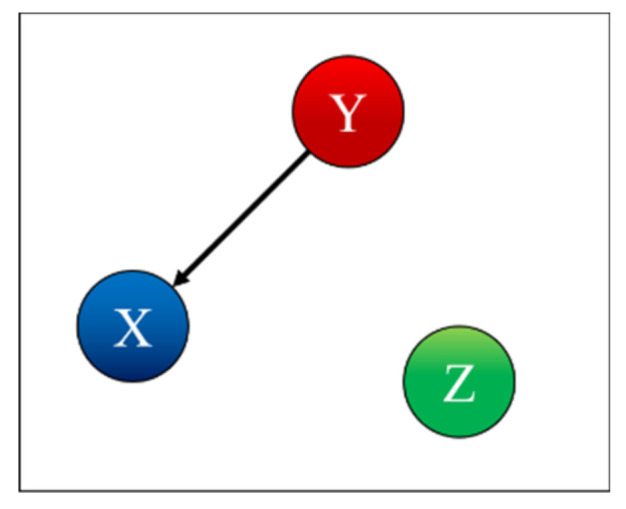
Coupling case 2.

Case 2 is the case where X is influenced by Y, as shown in Figure 5. Table 2 indicates correctly that X depends on X and is influenced by Y, Y depends only on Y, and Z depends only on Z. Indeed, removing the time series of the system Y, when predicting system X, rests in a miniscule probability of the null hypothesis being correct. Remembering that the null hypothesis is that of equal variances, its violation means that removing the input Y from the TDNNs causes a very high increase in the residuals of the X time series; therefore, Y contains important information about X and can be considered as causally related to X.

*Case 3: X is Influenced by Y and Z*

(12) $$\mu_{21}=\mu_{31}=0.1;\;\mu_{12}=\mu_{32}=\mu_{13}=\mu_{23}=0$$

**Figure 6:**
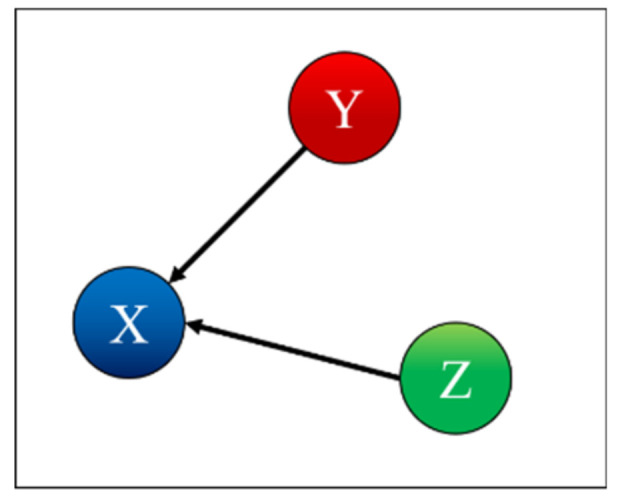
Coupling case 3.

Case 3 is the case where X is influenced by Y and Z, as shown in Figure 6. Table 3 indicates correctly that X depends only on X and is influenced by Y and Z, Y depends only on Y, and Z depends only on Z. Again, the interpretation of the table is that removing either the Y or Z inputs from the TDNNs causes the residuals to be significantly different, violating the null hypothesis of equal variance. The time series of Y and Z therefore carry information and can be assumed to be causally related to X.

*Case 4: X is Influenced by Y and Y is Influenced by Z*

(13) $$\mu_{21}=\mu_{32}=0.1;\;\mu_{12}=\mu_{31}=\mu_{13}=\mu_{23}=0$$

**Figure 7:**
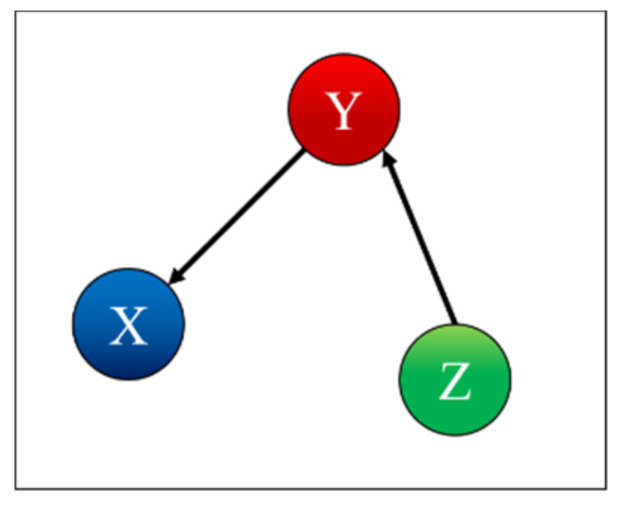
Coupling case 4.

Case 4 is the case where X is influenced by Y and Y is influenced by Z, as shown in Figure 7. Table 4 indicates correctly that X depends on X and is influenced by Y, Y depends on Y and is influenced by Z, and Z depends only on Z.

*Case 5: X is Influenced by Y and Z, Y is Influenced by Z*

(14) $$\mu_{21}=\mu_{31}=\mu_{32}=0.1;\;\mu_{12}=\mu_{13}=\mu_{23}=0$$

**Figure 8:**
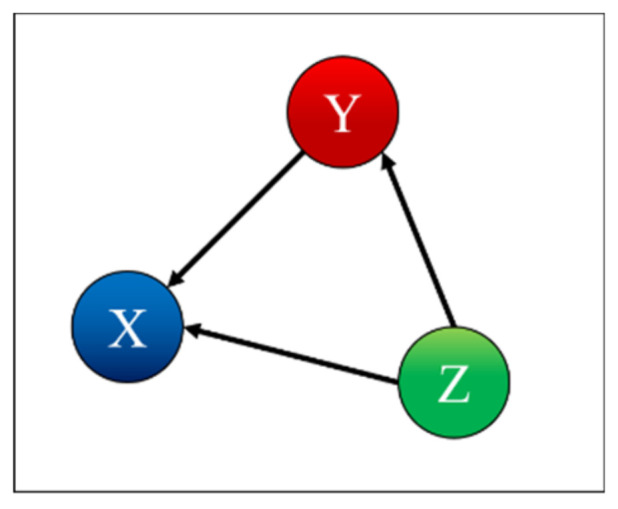
Coupling case 5.

In the case 5, both Y and Z influence X, and Y is influenced by Z, as shown in Figure 8. Table 5 indicates correctly that X depends on X and is influenced by Y and Z, Y depends on Y and is influenced by Z, and Z depends only on Z.

*Case 6: X is Influenced by Y, Y is Influenced by Z, and Z is Influenced by X*

(15) $$\mu_{21}=\mu_{32}=\mu_{13}=0.1;\;\mu_{12}=\mu_{31}=\mu_{23}=0$$

**Figure 9:**
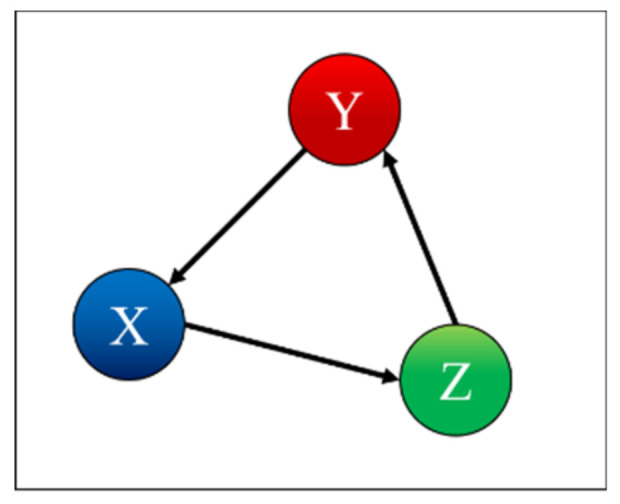
Coupling case 6.

Table 6 indicates correctly that X depends on X and is influenced by Y; Y depends on Y and is influenced by Z; Z depends on Z and is influenced by X, as shown in Figure 9. 

*Case 7: X is Influenced by Y and Z is Influenced by X.*

(16) $$\mu_{12}=\mu_{13}=0.1;\;\mu_{21}=\mu_{32}=\mu_{31}=\mu_{23}=0$$

**Figure 10:**
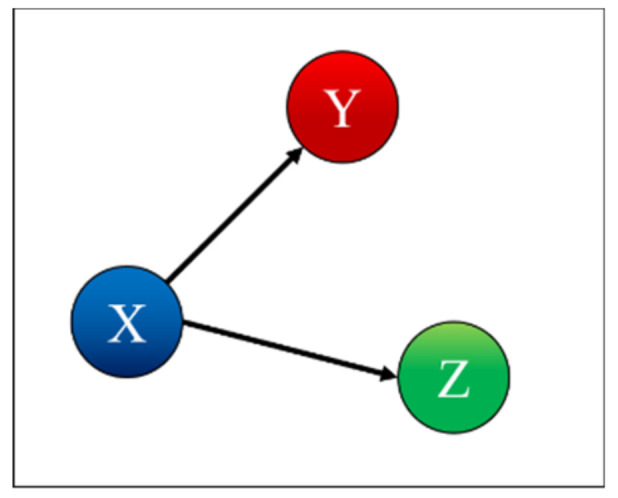
Coupling case 7.

Table 7 indicates correctly that X depends only on X, Y depends on Y and is influenced by X, and Z depends on Z and is influenced by X, as also shown in Figure 10.

To summarize, based on the F-test *p*-values of the residuals, it has always been possible to identify with great clarity the corrected influences, both direct and indirect, between the three systems X, Y and Z.

## 4. Time Localization of the Mutual Influence

To further investigate the potential of TDNNs, a preliminary analysis to assess the capability of this architecture to determine influences, which are localized in limited intervals of time, has been performed. To this end, case 4 (X influenced by Y, in its turn Y influence by Z) has been analyzed. The signals investigated are again those generated by the Lorenz systems discussed in Section 2. To localize the interactions in time, the mutual influence is modulated by changing the coupling coefficient (see later).

**Figure 11:**
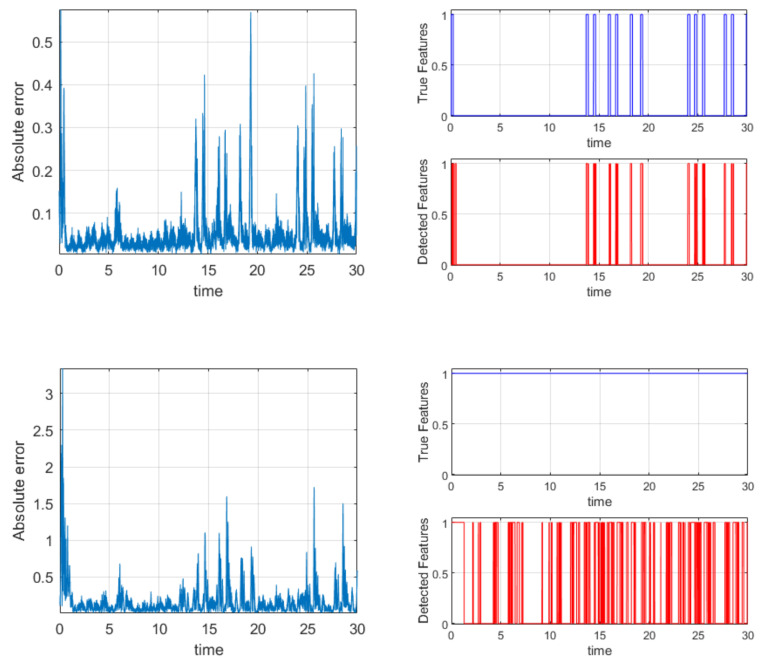
Top left: absolute error for the ZY interaction. Top right: modulation of the μ_32_ coupling coefficient above the detection of the coupling intervals by the TDNNs. Bottom left: absolute error for the YX interaction. Bottom right: constant μ_21_ coupling coefficient above the detection of the coupling intervals by the TDNNs (due to the amplitude variations of Y).

As expected, the TDNNs correctly identify the influences between the time series over the entire interval analyzed. To perform a time resolved analysis, the following quantity has been defined:

(17) $$Absolute\;Error\left(t\right)=\sqrt{\left(Err_{wt}^{2}\left(t\right)-Err_{all}^{2}\left(t\right)\right)}$$

where *Err_all_* indicates the total root square error in the prediction when all the signals are used as inputs, and *Err_wt_* the total error when one of the time series has been deselected from the input list (there are therefore three different *Err_wt_*). A time series is considered as influencing another when:

(18) $$Detected\;Features\left(t\right)=\frac{Absolute\;Error-median\left(Err_{all}\right)}{std\left(Err_{all}\right)}>Z_{threshold}$$

in which *Z_threshold_* is the *Z*-score, set at a value of two for the cases reported in the following. The plots in Figure 11 show the results obtained for one of the most important cases analyzed (case 4).

The influence between Z and Y has been modulated; the values of μ_32_ have been shifted abruptly from 0 to 1 at every time slice, in which Z_2_ is higher than 10 (see Figure 11 top plot on the right). The absolute error follows the same trend of the coupling coefficient, and the mutual influence is detected in the right intervals (again see Figure 11 top plots). The mutual coupling between Y and X has been kept constant at a value of 1. This situation allows detecting when Y has a sufficient amplitude to really influence X. Indeed, it can be seen from the absolute errors that when Y has a minuscule amplitude, it cannot exert any influence on X, even if the coupling coefficient is 1. Therefore, the oscillations in the bottom right plot of Figure 11 accurately reflect the actual evolution of the real mutual influence between the systems.

To conclude, the tests performed, and exemplified by the case reported, have provided very good results. The TDNNs can identify the right time intervals in which the mutual influence is active, both in the case of modulation of the coupling coefficient and oscillations of the driver amplitude.

## 5. First Analysis of Noise Effects

Encouraging preliminary results have also been achieved in the analysis of the effects of additive noise. For the same case as before, case 4 of Z influencing and Y influencing X has been investigated; random noise of Gaussian distribution has been added to the time series. The noise is centered around zero, and the standard deviation I_noise_ has been scanned over a wide range. Table 8 shows how the TDNNs properly determined the causal relationships between the three systems X, Y, and Z, except for the cases in which the noise becomes excessive. Basically, provided the signal of noise ratio is higher than 2, the TDNNs always correctly identify the causal relationships between the time series. A more systematic analysis of the noise influence will have to be carried out, but the first indications are very encouraging, and there is no reason to expect that they will not be confirmed in the future.

## 6. Conclusions

A specific topology of time delay neural networks has been devised for the exhaustive investigation of the direct and indirect coupling between three time series. The time series have been generated by three Lorenz systems in the chaotic regime. The TDNNs always manage to properly identify the real couplings even with inputs of only two delayed times and a very limited number of examples. In addition to the accuracy, the capability to operate with sparse data is another very important upside of the proposed network architecture. Various tests also show the potential of the networks to identify the actual time localization in the mutual influence between the systems. Preliminary indications about the TDNNs capability to handle significant levels of noise are also very positive. To conclude the summary of the performance, a comment is in place about the reproducibility of the networks. As is well known, due to the random aspects of network training, the outputs of TDNNs are not fully deterministic. Even with the same inputs, the outputs can be slightly different. In the present type of application, this problem can be easily remedied. One alternative is the choice of strict constraints on the parameters of the networks (number of iterations, maximum tolerated error, minimum gradient, etc.). Probably a more reliable solution consists of repeating the analysis a certain number of times and then draw the conclusions based on a suitable decision function (typically some form of majority voting is more than adequate).

In terms of future developments, it is planned to apply the proposed methodology to other classes of systems, particularly those with significant memory effects. A more systematic analysis of the influence of various noise statistics is also to be carried out. After completely documenting the properties of the proposed TDNNs, a careful comparison of their performances, with other techniques reported in the literature for the investigation of the mutual influence between time series, is also planned. Specific attention will be granted to Bayesian methods, which can associate robust uncertainty quantification to their predictions, something of great relevance for scientific applications. Moreover, also alternative approaches to neural computing and learning, for example of the type reported in [12,13], will be carefully considered. In any case, from a preliminary comparison of the results presented in this paper with those reported in the literature, it seems that the TDNNs, with two hidden layers, could prove to be very competitive. It should be mentioned that a single hidden layer has proved to be adequate only for simple linear interactions. Already for the non-linearities considered in the paper, two hidden layers are essential to obtain good results. For more complex forms of non-linear interactions, even more layers could be necessary. The two-layer topology is indeed the basic architecture used also in [14] for the Nonlinear Autoregressive Exogenous Model (NARX) model. The main difference between the two works resides mainly in the final objective; in the present study, the goal is the determination of the causal relationship between different time series, while in [14], long-term recursive prediction is the main topic of interest. Therefore, one-step-ahead prediction is adequate for the present work, whereas longer future forecasting required a different training approach in the NARX model.

With regard to future applications, the proposed networks are expected to become very useful for the analysis of complex systems, particularly in the field of thermonuclear fusion [15,16,17,18,19,20,21], possibly in combination with new metrics to analyze the residuals [22,23,24]. More generally, the analysis of non-conventional events in many industrial environments and even security contexts constitute another interesting sector of potential applications [25].

## Figures and Tables

**Table 1:** F-Test *p*-Value.

		Removed Variable		
		*X*	*Y*	*Z*
**Predicted**	*X*	0.00%	99.82%	97.46%
	*Y*	81.01%	0.00%	40.84%
	*Z*	74.24%	44.57%	0.00%

**Table 2:** F-Test *p*-Value.

		Removed Variable		
		*X*	*Y*	*Z*
**Predicted**	*X*	0.00%	3.97E-07	88.17%
	*Y*	27.57%	0.00%	7.89%
	*Z*	80.36%	40.01%	0.00%

**Table 3:** F-Test *p*-Value.

		Removed Variable		
		*X*	*Y*	*Z*
**Predicted**	*X*	0.00%	4.55E-09	4.23E-55
	*Y*	68.33%	0.00%	67.01%
	*Z*	10.34%	54.85%	0.00%

**Table 4:** F-Test *p*-Value.

		Removed Variable		
		*X*	*Y*	*Z*
**Predicted**	*X*	0.00%	0.00%	73.18%
	*Y*	84.94%	0.00%	0.00%
	*Z*	40.19%	74.63%	0.00%

**Table 5:** F-Test *p*-Value.

		Removed Variable		
		*X*	*Y*	*Z*
**Predicted**	*X*	0.00%	1.16%	0.00%
	*Y*	96.52%	0.00%	0.00%
	*Z*	61.21%	59.18%	0.00%

**Table 6:** F-Test *p*-Value.

		Removed Variable		
		*X*	*Y*	*Z*
**Predicted**	*X*	0.00%	0.00%	82.33%
	*Y*	48.62%	0.00%	0.00%
	*Z*	0.00%	58.31%	0.00%

**Table 7:** F-test *p*-Value.

		Removed Variable		
		*X*	*Y*	*Z*
**Predicted**	*X*	0.00%	24.81%	30.65%
	*Y*	0.00%	0.00%	16.03%
	*Z*	0.00%	60.72%	0.00%

**Table 8:** Causal Relationships for Case 4 of Figure 1.

I_noise_	Mean SNR	Z to Z	Z to Y	Z to X	Y to Z	Y to Y	Y to X	X to Z	X to Y	X to X
0.01	30	1	1	0	0	1	1	0	0	1
0.02	16	1	1	0	0	1	1	0	0	1
0.05	7	1	1	0	0	1	1	0	0	1
0.1	3	1	1	0	0	1	1	0	0	1
0.2	1.2	1	1	0	0	1	1	0	0	1
0.5	0.4	1	0	0	0	1	1	0	0	1
1	0.2	1	0	0	0	1	0	0	0	1
Expected		1	1	0	0	1	1	0	0	1
